# Interferon receptor gene dosage differentially regulates hypoxia-induced platelet activation and pulmonary hypertension in down syndrome

**DOI:** 10.3389/fimmu.2026.1832057

**Published:** 2026-05-22

**Authors:** Janelle N. Posey, Mariah Jordan, Amanda Olsen-Dufour, Thi-Tina N. Nguyen, Christine Farrell, Caitlin V. Lewis, Jamie L. Archambault, Christina Sul, Daniel Colon-Hidalgo, Eva S. Nozik, Joaquin M. Espinosa, Kelly D. Sullivan, Cassidy Delaney

**Affiliations:** 1 Section of Neonatology, Department of Pediatrics, University of Colorado Anschutz, Aurora, CO, United States; 2 Cardiovascular Pulmonary Research Laboratories, University of Colorado Anschutz, Aurora, CO, United States; 3 School of Medicine, University of Colorado Anschutz, Aurora, CO, United States; 4 Section of Pediatric Critical Care, Department of Pediatrics, University of Colorado Anschutz, Aurora, CO, United States; 5 Division of Pulmonary Sciences and Critical Care Medicine, Department of Medicine, University of Colorado Anschutz, Aurora, CO, United States; 6 Linda Crnic Institute for Down Syndrome, University of Colorado Anschutz, Aurora CO, United States; 7 Department of Pharmacology, University of Colorado Anschutz, Aurora, CO, United States; 8 Section of Developmental Biology, Department of Pediatrics, University of Colorado Anschutz, Aurora, CO, United States

**Keywords:** hypoxia, interferon, platelet, pulmonary hypertension, thromboinflammation, trisomy 21, vascular injury

## Abstract

Trisomy 21 (T21) results in Down syndrome (DS), a condition associated with a high prevalence of pulmonary complications. Pulmonary hypertension (PH) is a significant comorbidity in individuals with T21. Inflammation is a well-established driver of PH, and growing evidence implicates platelets as active contributors to inflammatory-mediated pulmonary vascular disease and PH. In T21, increased interferon (IFN) signaling resulting from an additional IFN receptor gene locus contributes to immune dysregulation, and interactions between platelet activation and IFN signaling may promote thromboinflammatory pathways associated with vascular disease. To determine whether platelet activation is altered in T21, we first measured platelet activation by flow cytometry in individuals with T21 and age-matched controls. We then utilized the Dp16 mouse model of down syndrome to investigate IFN-dependent mechanisms. WT, Dp16, and Dp162xIfnrs mice 7-9 weeks of age were exposed to 10% hypobaric hypoxia for 3 or 21 days or remained at Denver altitude. Platelet activation was measured by flow cytometry. Lungs were collected to measure PF4 by ELISA and lung platelets by IHC. Vascular remodeling was measured by IHC staining of muscularized small vessels. PH was measured by RSVP and RV hypertrophy (RVH). Baseline and agonist-induced platelet activation were increased in individuals with T21, as evidenced by elevated platelet P-selectin expression and activated αIIbβ3. To investigate the immunoregulatory mechanisms underlying T21-associated PH, we utilized the Dp16 mouse model of Down syndrome in a chronic hypoxia model of PH. Consistent with our human findings, baseline, agonist-induced, and hypoxia-induced P-selectin and αIIbβ3 activation were elevated in Dp16 mice compared with wild-type (WT). Circulating PF4 and soluble GPVI were elevated at baseline and PF4 was further increased in hypoxic Dp16 mice. Lung PF4 levels were also increased at baseline and further elevated with hypoxia in Dp16 mice, despite comparable numbers of lung platelets. Dp16 mice demonstrated increased baseline pulmonary vascular remodeling, right ventricular systolic pressure (RVSP), and right ventricular hypertrophy (RVH) with both RVSP and RVH being further exacerbated in hypoxic Dp16 mice. Normalization of *Ifnr* copy number attenuated hypoxia-induced platelet activation and lung PF4 accumulation. Normalization of *Ifnr* copy number corrected the baseline increase in pulmonary vascular remodeling and restored RVSP to WT levels. RVH remained elevated at baseline and following hypoxia in Dp16_2xIfnrs_ mice, indicating that *IFNR* signaling does not fully account for cardiopulmonary phenotypes associated with PH. Together, these findings demonstrate that IFN signaling contributes to platelet activation and pulmonary vascular remodeling in Dp16 mice, but does not drive PH severity, suggesting additional mechanisms in the development of T21-associated PH. This work provides novel insight into the role of IFN signaling and platelet activation in Down syndrome -associated pulmonary vascular disease and has potential clinical relevance given the presence of platelet activation in individuals with T21.

## Introduction

Pulmonary hypertension (PH) is characterized by aberrant pulmonary vascular remodeling resulting in a mean pulmonary artery pressure > 20 mmHg and pulmonary vascular resistance of ≥ 2 Wood units (1–5). Even with therapeutic advancements, patients with PH face poor survival outcomes, with a median survival of 5–10 years following diagnosis (1, 2, 6, 7). PH is increasingly recognized as an immune-mediated disease and we and others have highlighted the role of platelets as key mediators in this process (8–14). Accumulating mechanistic studies indicate that platelet activation is not merely a secondary consequence of PH, but actively contributes to disease initiation and progression by promoting PVR, inflammation, and thrombosis (5, 15–24). Platelet activation and accumulation in the lung have been observed clinically and in experimental models of PH and numerous platelet-derived proteins, such as chemokine platelet factor 4 (PF4), have been found at elevated levels in both the pulmonary vasculature and circulation in patients with PH (9, 15–17, 20, 23, 25–29). PF4 contributes to inflammation and vascular remodeling in PH by recruiting immune cells to the lung and stimulating hyperplasia and vessel wall thickening (22, 30–33).

Down syndrome or Trisomy 21 (T21), is the most common chromosomal disorder in humans, affecting approximately 1 in 700 live births (34–36). Although survival rates have improved over time, individuals with T21 have a median life expectancy roughly three decades shorter than average, with significant cardiopulmonary morbidity and mortality (34, 35, 37). PH is a significant comorbidity in individuals with T21, with an estimated lifetime incidence of 10-20% (36, 38). Alterations in platelet function and biology, including changes in platelet size, count, and granule content, have also been observed in T21, potentially influencing the progression of PH (39–41). Despite therapeutic progress, the underlying mechanisms driving PH in this population remain incompletely understood, highlighting the need for continued mechanistic research to develop targeted and innovative treatment strategies (42, 43).

T21 causes triplication and overexpression of genes located on human chromosome 21, including a cluster of four interferon receptor (IFNR) genes: *IFNAR1*, *IFNAR2*, *IFNGR2*, and *IL10RB*. Type I IFNs (IFN-α, IFN-β), type II IFNs (IFN-γ), and type III IFNs (IFN-λ) signal through these receptors and are central to innate immune activation (44, 45). This overexpression of IFNR*s* causes interferonopathy, a chronic state of heightened interferon (IFN) signaling that contributes to systemic inflammation and immune dysregulation, resulting in increased susceptibility to autoimmune conditions and more severe complications from infections (34, 36, 37, 42, 44–51). IFN therapies have been associated with the subsequent development of PH in humans and murine models have demonstrated that genetic deletion of IFNRs attenuates hypoxia-induced PH which suggests a potential mechanistic link between the triplication of four IFNR genes in T21 and PH (47, 52). Interferon signaling has been shown to directly modulate platelet activation, including recent evidence demonstrating interferon-driven thrombotic priming of platelets in inflammatory disease (51, 53). Platelets act as both targets and amplifiers of IFN signaling, releasing pro-inflammatory and vasoactive mediators that influence endothelial and smooth muscle cell function (5, 54–61). Together, these factors underscore the potential interplay between IFN signaling, platelet activation, and vascular remodeling in the development of PH in individuals with T21.

The Dp(16)1Yey (Dp16) mouse is a widely used model of Down Syndrome that houses a segmental duplication on chromosome 16, resulting in the triplication of approximately 120 protein-coding genes orthologous to those on human chromosome 21, including the *Ifnr* cluster (49). Dp16 mice recapitulate key immunologic and inflammatory features of T21, including constitutive IFN hyperactivity and chronic systemic inflammation, making them an appropriate model to study IFNR-driven mechanisms in Down Syndrome (49). Normalization of *Ifnr* copy number in Dp16 mice prevents congenital heart malformations and developmental delays and improves cognitive functioning (45). Additionally, Dp16 mice have IFN dependent exacerbation of immune responses and lethal immune hypersensitivity to viral mimetics, which are ameliorated by both genetic normalization of *Ifnr* copy number and pharmacological blockade of downstream JAK/STAT signaling (45, 50).

Given the constitutive interferonopathy of T21 and its potential downstream effects on platelet biology, we hypothesized that dysregulated IFN signaling contributes to the pathogenesis of PH in individuals with T21 and in Dp16 mice. We hypothesized that in the Dp16 mouse model, platelet activation and PH are heightened both at baseline and in response to hypoxic stress as a consequence of chronic inflammation. To assess the IFN dependence of these responses, we utilized a novel genetically modified Dp16 mouse with normalized *Ifnr* copy number (Dp16^2xIfnrs^) described in **Supplementary Figure 1A**. Elucidating these mechanisms may provide critical insight into disease pathogenesis and offer new therapeutic opportunities for managing PH in individuals with T21.

## Methods

### Preparation of human blood and platelets

Study participants were recruited to the Human Trisome Project, a large cohort study of the population with Down syndrome approved by the Colorado Multiple Institutional Review Board (COMIRB #15-2170) and conducted in accordance with the Declaration of Helsinki. Written informed consent was obtained from all participants or their legal guardians. Study participants include male and female donors. Participant characteristics, including age, sex, and body mass index (BMI), are summarized in **Table 1**. All participants were obtained as a convenience sample of volunteer donors, and clinical data were limited to available demographic characteristics. Importantly, no participants in either group had a diagnosis of pulmonary hypertension (PH). It was confirmed that donors had not taken antiplatelet or anticoagulant drugs, nonsteroidal anti-inflammatory drugs, or consumed alcohol in the prior 48 hours, and were not pregnant at the time of blood donation. Blood was collected by venipuncture using a 22-gauge needle into EDTA Vacutainer tubes. The first 4.0 mL of blood was discarded. EDTA-anticoagulated whole blood was used to obtain CBCs using the Heska HT5 hematologic Analyzer (Loveland, Colorado, USA) and processed for platelet activation.

**Table 1:** Participant characteristics.

Characteristic	Control N = 7	T21 N = 5	Overall N = 12	p-value
Age, years	26.6 (26.5, 32.1)	29.4 (27.2, 37.5)	27.0 (26.6, 34.8)	0.515
Sex				0.558
Female	5 (71%)	2 (40%)	7 (58%)	
Male	2 (29%)	3 (60%)	5 (42%)	
Race				>0.999
Other / Multiracial	2 (29%)	1 (20%)	3 (25%)	
White	5 (71%)	4 (80%)	9 (75%)	
Ethnicity				>0.999
Hispanic or Latino	2 (29%)	1 (20%)	3 (25%)	
Not Hispanic or Latino	5 (71%)	4 (80%)	9 (75%)	
BMI category				0.072
Underweight	0 (0%)	0 (0%)	0 (0%)	
Normal weight	6 (86%)	1 (20%)	7 (58%)	
Overweight	1 (14%)	4 (80%)	5 (42%)	

All participants were obtained as a convenience sample of volunteer donors, and clinical data were limited to available demographic variables. No participants in either group had a diagnosis of pulmonary hypertension (PH).

1. Median (Q1, Q3); n (%).

2. Wilcoxon rank sum test; Fisher’s exact test.

BMI categories were defined as underweight (<18.5 kg/m²), normal weight (18.5–24.9 kg/m²), overweight (25.0–29.9 kg/m²), and obesity (≥30.0 kg/m²). Continuous variables are presented as median (IQR), and categorical variables are presented as n (%). Group comparisons were performed using the Wilcoxon rank-sum test for continuous variables and Fisher’s exact test for categorical variables.

### Assessment of human platelet activation

EDTA-anticoagulated whole blood was centrifuged at 100g for 20 minutes at RT. Platelet-rich plasma was collected, transferred to a new tube, and supplemented with PGI_2_ (1 μg/mL; Cayman Chemical; Ann Arbor, Michigan, USA) and apyrase (0.02 U/mL; Sigma-Aldrich; Burlington, Massachusetts, USA) and incubated for 3 minutes at RT to prevent pre-activation and centrifuged at 2000g for 5 minutes. The supernatant was discarded and the platelet pellet was resuspended in 1 mL of warm Modified Tyrode’s buffer (pH 7.3; [NaCl 129 mM, KCl 2.9 mM, MgCl_2–_1 mM, NaH_2_PO_4_ 0.34, NaHCO_3_ 12, and Glucose 5mM]). Washed platelets (platelet count, 1 × 10^6^/mL) were diluted in 100 μL of warm Tyrode’s buffer containing 1mM CaCl_2_. Platelets were activated for 20 minutes at 37 C in the dark with thrombin (0.01 U/mL; Chrono-log; Havertown, Pennsylvania, USA), in the presence of anti-human CD41-BV421 antibody (clone no. HIP8; 1:20; BioLegend, San Diego, CA, USA), anti-mouse/human P-selectin–APC (clone no. APM-1; 1:25; BioLegend, San Diego, CA, USA), and PAC-1-PE (activated integrin αIIbβ3) antibody (clone no. A2A9/6; BioLegend, San Diego, CA, USA). Data was analyzed using Kaluza Analysis Software (v2.3, Beckman Coulter, Brea, California, USA). Blinding was not feasible for human samples. Platelets were differentiated from WBCs and debris by size discrimination using FSC and SSC and were defined as CD41+ singlets. To classify activated platelets, 50,000 platelets were captured and analyzed for P-selectin and activated integrin αIIbβ3 expression. Unstained and fluorescence-minus-one controls were used to determine monoclonal antibody (mAb) gating and data is expressed as a percentage of positive cells in the target gate.

### Mouse model

Experiments were approved by the Institutional Animal Care and Use Committee at the University of Colorado Anschutz Medical Campus under protocol 00520 and performed in accordance with National Institutes of Health (NIH) guidelines. Mice were obtained from Dr. Joaquin Espinosa (University of Colorado Anschutz, Department of Pharmacology, Linda Crnic Institute for Down Syndrome). “This model has been genetically validated using fluorescence *in situ* hybridization (FISH), array comparative genomic hybridization (aCGH), and quantitative PCR in prior studies (49, 62, 63). Wild-type (WT) and Dp16 mice were originally obtained from The Jackson Laboratory (Bar Harbor, ME), and genotypes were confirmed using established PCR-based genotyping protocols (62). Heterozygous WT^1xIfnr^ mutant mice were generated in collaboration with Dr. Jennifer Matsuda and James Gross (Genetics Core Facility, National Jewish Health, CO). Female WT^1xIfnrs^ were intercrossed with Dp(16)1Yey/+ (Dp16) males to generate WT, Dp16, and Dp16^2xIfnrs^ mice as previously described (45, 49). Dp16^2xIfnrs^ mice maintain the MMU16 segmental duplication while carrying a heterozygous deletion of the *Ifnr* gene cluster, thereby normalizing interferon receptor gene dosage (**Supplementary Figure 1A**). This model recapitulates key molecular and immunological features of trisomy 21, including interferon pathway dysregulation relevant to the present study (45, 48, 49, 64). All mice were bred at Denver altitude and are viable and fertile. Mice were evaluated at 10–15 weeks of age and had no differences in body weight (**Supplementary Figure 1B**). Control groups remained at Denver altitude, while experimental groups were exposed to hypobaric hypoxia (PB=380 mmHg, FiO_2_ = 10%) for either 3 or 21 days. The 3-day exposure was selected to capture early responses to hypoxic stress, including inflammatory signaling and platelet activation. The 21-day exposure represents a well-established timepoint for the development of pulmonary hypertension phenotypes in the chronic hypoxia model, including increased right ventricular systolic pressure, right ventricular hypertrophy, and pulmonary vascular remodeling, as previously described (**Supplementary Figure 2**) (28, 32, 65–68).

### Preparation of murine blood, platelets, and plasma

Mice were anesthetized with 2% isoflurane, and blood was obtained via terminal closed-chest cardiac puncture using heparin or acid-citrate dextrose (ACD) containing syringes (1:6) and processed for platelet activation. Heparinized whole blood was used to obtain CBCs using the Heska HT5 hematologic Analyzer (Loveland, Colorado, USA). Platelet-rich plasma (PRP) was separated by centrifugation of heparinized whole blood at 100g x 16 minutes. PRP was supplemented with PGI_2_ (1ug/mL, Cayman Chemical; Ann Arbor, Michigan, USA) and apyrase (0.02U/mL, Sigma-Aldrich; Burlington, Massachusetts, USA) to avoid platelet pre-activation and incubated at room temperature (RT) for 3 minutes before centrifugation at 2000g × 2 minutes to obtain platelet-poor plasma (PPP). Plasma samples were stored at -80C until use.

### Assessment of murine platelet activation

Acid citrate dextrose (ACD) anticoagulated blood was diluted with 1mL of warm Modified Tyrode’s Buffer Modified Tyrode’s buffer (pH 7.3; [NaCl 129 mM, KCl 2.9 mM, MgCl_2–_1 mM, NaH_2_PO_4_ 0.34, NaHCO_3_ 12, and Glucose 5mM]) containing PGI_2_ (1ug/mL) and apyrase (0.02U/mL) to avoid platelet pre-activation and incubated for 3 minutes at RT. Diluted blood was centrifuged at 150g x 5 minutes at RT. Platelet-rich plasma (PRP) was collected, transferred to a new tube with PGI_2_ (1ug/mL) and apyrase (0.02U/mL), and incubated for 3 minutes (RT). Platelets were pelleted at 450g x 10 minutes (RT), and the supernatant was discarded. Platelet pellets were resuspended in 500μL of warm Tyrode’s Buffer and diluted (1 × 10 (6) platelets) in 100μL of warm Tyrode’s buffer containing 1mM CaCl_2._ Platelets were incubated for 10 minutes at RT in the dark in the presence of the of anti-mouse CD41-BV421 antibody (Clone No. MWReg30 1:50; BioLegend, San Diego, CA, USA), P-selectin-APC (Clone No. Psel.KO2.3, 1:25; Thermofisher, Denver, CO, USA), and activated integrin αIIbβ3-PE antibody (Clone No. JON/A, 1:20; Emfret, Würzburg, Germany) in the presence or absence of agonist thrombin (Chrono-log Corporation, Havertown, PA, USA). The assay was quenched at 10 minutes by diluting the blood 1:5 using RT 1% PFA in Tyrode’s Buffer. Data was analyzed by a blinded investigator using Kaluza Analysis Software (v2.3, Beckman Coulter, Brea, California, USA). Platelets were differentiated from WBCs and debris by size discrimination using FSC and SSC and were defined as CD41+ singlets. To classify activated platelets, 50,000 platelets were captured and analyzed for P-selectin and activated integrin αIIbβ3 expression. Unstained and fluorescence-minus-one controls were used to determine monoclonal antibody (mAb) gating and data is expressed as a percentage of positive cells in the target gate.

### PF4 ELISA

Platelet-poor plasma was obtained as described above and frozen at -80C. Lungs were flushed as described above and collected in AllProtect Tissue Reagent (Qiagen, Germantown, MD, USA). Tissues were lysed in Pierce’s Tissue Protein Extraction Reagent containing Sigma’s Phosphatase Inhibitor Cocktail 2, Sigma’s Phosphatase Inhibitor Cocktail 3, and Sigma’s Protease Inhibitor Cocktail per manufacturer’s instructions (Sigma, St Louis, MO, USA). Lung homogenates were prepared from 25-30mg of lung tissue in prepared tissue lysis buffer using the Bead Ruptor_12_ (Omni International, Kennesaw, GA, USA), as previously described (32). Homogenized samples were incubated for 30 minutes on ice, then centrifuged at 10,000g for 5 minutes and stored at -80C. PF4 was measured in platelet-poor plasma and lung homogenates using the mouse PF4 ELISA kit (ab100735; Abcam, Cambridge, Massachusetts, USA).

### sGPVI ELISA

Platelet-poor plasma was obtained as described above and frozen at -80C. Soluble GPVI was measured in platelet-poor plasma using the mouse GPVI ELISA kit (OKEH05215; Aviva Systems Biology Corporation, San Diego, California, USA).

### Evaluation of lung platelets

Lungs were flushed as described above and inflated with 4% paraformaldehyde for paraffin embedding and immunohistochemistry was performed for CD41. IHC staining and quantification were performed on lung sections using anti-mouse CD41 (1:200, GTX113758; Genetex, Irvine, California, USA) and Dako EnVision + Dual Link System-anti-rabbit HRP DAB+ detecting system (Agilent, Carpinteria, California, USA) as previously described (28, 32, 65). Briefly, whole lung scans obtained using the Leica Aperio VERSA brightfield scope (40x objective, 0.5-micron resolution). Ten random images per slide were captured, excluding lung fields with large vessels or airways, and distal lung platelets were assessed by quantifying CD41+ pixels per high-powered field (×20. We used a random, unbiased approach with ImageScope, version 12.4.3.500 (Leica Biosystems Imaging, Inc., Deer Park, Illinois, USA), pixel-quantification software. An investigator blinded to the experimental groups performed the analysis.

### Evaluation of muscularized vessel density

Pulmonary vascular remodeling was assessed by evaluating muscularized vessel density. Mouse lungs were flushed with 10mL of phosphate buffered saline (PBS) and inflated for paraffin embedding, and immunohistochemistry (IHC) was performed for α-smooth muscle actin (α-SMA) as previously described (32). Briefly, IHC staining and quantification of muscularized vessels were performed on lung sections using anti-mouse α-SMA (1:1200, A2547; Sigma, St. Louis, Missouri, USA) and Mouse on Mouse (M.O.M.) immunodetection kit (Vector, Burlingame, California, USA) as previously described (67, 69). Briefly, slides were developed with DAB+ and counterstained with methyl green (Statlab, McKinney, Texas, USA). Whole slide scans were collected using the Leica Aperio VERSA brightfield scope (40x objective, 0.5-micron resolution). Ten random images per slide excluding lung fields containing large vessels or airways were captured. Muscularization of small vessels was assessed by counting the number of α-SMA-positive vessels <50 µm per high-power field (x10). An investigator blinded to the experimental group performed the analysis.

### Hemodynamic measurements and evaluation of RVH

PH was assessed by right ventricular systolic pressures (RVSPs) and right ventricular hypertrophy (RVH) measured at 21 days. RVSP was obtained by closed-chest right ventricle (RV) puncture using AcqKnowledge, version 3.9.1-100M (BIOPAC Systems, Inc. Goleta, California, USA). Reported values are obtained from an average of 3-second RV waveforms. RVH was measured by manually dissecting the heart to isolate the free wall of the right ventricle (RV) from the left ventricle (LV) and septum (S) to obtain Fulton’s index, or the ratio of RV weight over LV+S weight (RV/LV+S). An investigator blinded to the experimental group performed the analysis.

### Antibody validation

All antibodies have been previously validated in our laboratory using positive and negative controls (21, 28, 32, 65, 70, 71).

### Statistical analysis

Normality was assessed using the Shapiro–Wilk test. Statistical analyses for parametric analyses were performed using GraphPad Prism (v11.0.0; GraphPad Software, La Jolla, California, USA). Group comparisons were conducted using Student’s *t*-test or two-way ANOVA followed by Tukey’s *post hoc* test for multiple comparisons, as appropriate. When assumptions of normality were not met, aligned rank transformation ANOVA was performed, followed by pairwise comparisons using estimated marginal means with Holm adjustment implemented in RStudio (v4.5.3, RStudio: Integrated Development Environment for R, Boston, Massachusetts, USA). Outlier testing was applied uniformly across all groups and assessed using the interquartile range method within genotype-by-condition groups and by a robust regression–based false discovery rate approach analogous to ROUT (Q=1%). Statistical significance was defined as p ≤ 0.05. Data are presented as mean ± SEM.

## Results

### Baseline and agonist-induced platelet activation are increased in individuals with T21

**Figure 1:**
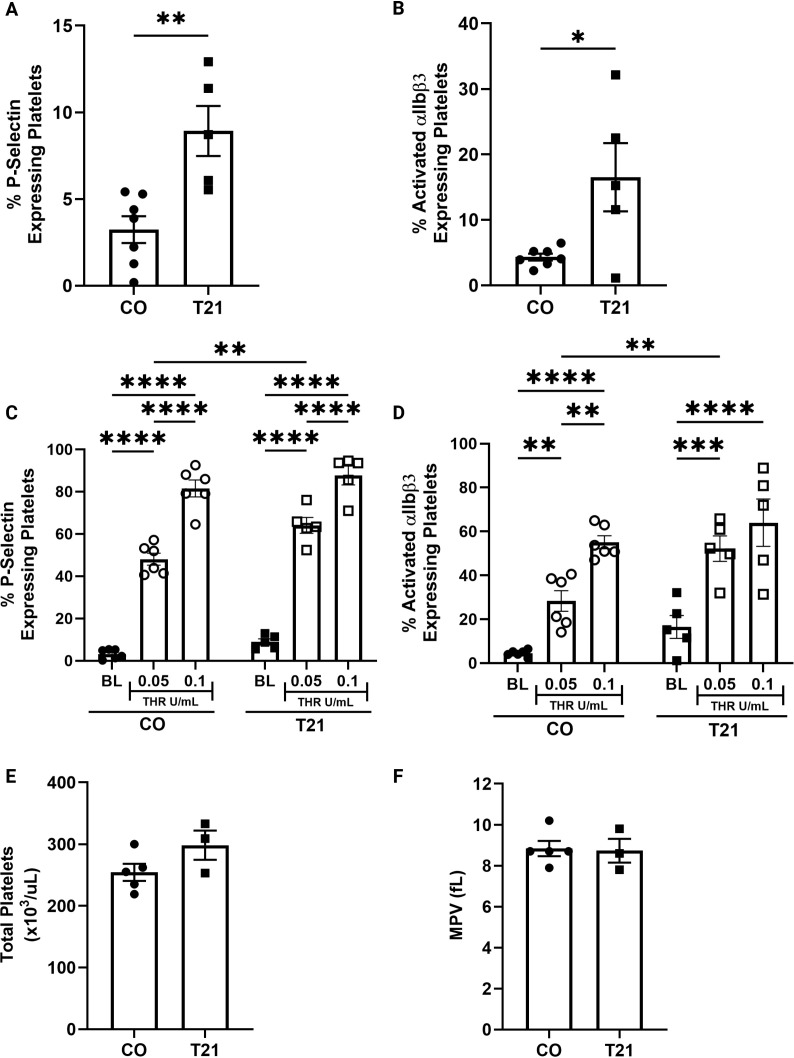
Baseline and agonist-induced platelet activation are increased in patients with T21. Platelet activation measured in human donors. *CO, n=7; T21, n=5.* **(A, B)** The percentage of P-selectin and activated αIIbβ3 (PAC-1) expressing platelets is elevated in patients with Trisomy 21 compared to matched controls. Activation potential was measured in platelets stimulated with Thrombin. *CO, n=6; T21, n=5.* **(C, D)** Moderate dose agonist-induced P-selectin release and αIIbβ3 (PAC-1) activation is exacerbated in patients with Trisomy 21 compared to matched controls. High dose agonist induced platelet activation is similar between groups. **(E, F)** Platelet count and mean platelet volume (MPV) in human donors. *CO, n=5; T21, n=3.* Total circulating platelet count and MPV are similar in patients with T21 and matched controls. Data were analyzed using unpaired Student’s t-tests for two-group comparisons **(A, B, E, F)** and two-way ANOVA with Tukey’s *post hoc* testing for agonist conditions **(C, D)**. Sample sizes vary by panel due to availability of complete datasets for each assay. All data are presented as mean ± SEM. Statistics: *p ≤ 0.05, **p<0.01, ***p<0.001, ****p<0.0001.

Chronic low-grade inflammation is a hallmark of T21, arising from aberrant activation and signaling across multiple immune cell types that sustain immune dysregulation (46, 48, 50, 72–74). Given the potential role of platelet-mediated thromboinflammation in pulmonary vascular disease, we first assessed platelet activation in individuals with T21. Baseline platelet activation was increased in individuals with T21 compared to matched controls, as evidenced by elevated surface P-selectin expression and increased activation of integrin αIIbβ3 (**Figures 1A, B**). To evaluate activation potential, platelets were stimulated with thrombin. Moderate-dose thrombin induced increased P-selectin and αIIbβ3 activation in T21 platelets compared to matched controls indicating an enhanced response to stimulation. High-dose thrombin resulted in similar activation across groups, confirming that T21 platelets are capable of maximal activation (**Figures 1C, D**). Lastly, we found no differences in platelet count or mean platelet volume (MPV) between individuals with T21 and matched controls (**Figures 1E, F**). There were no differences in age, sex, or BMI between groups, though there is a moderate correlation between BMI and platelet activation (Pearson’s r=0.59, p=0.04; **Table 1**). None of the participants had a diagnosis of PH at the time of this study. These findings should be interpreted as phenotypic proof-of-principle, as this cohort was exploratory and not designed to assess pulmonary hypertension status or disease severity.

### Platelet activation is increased and *Ifnr* copy-dependent in Dp16 mice

**Figure 2:**
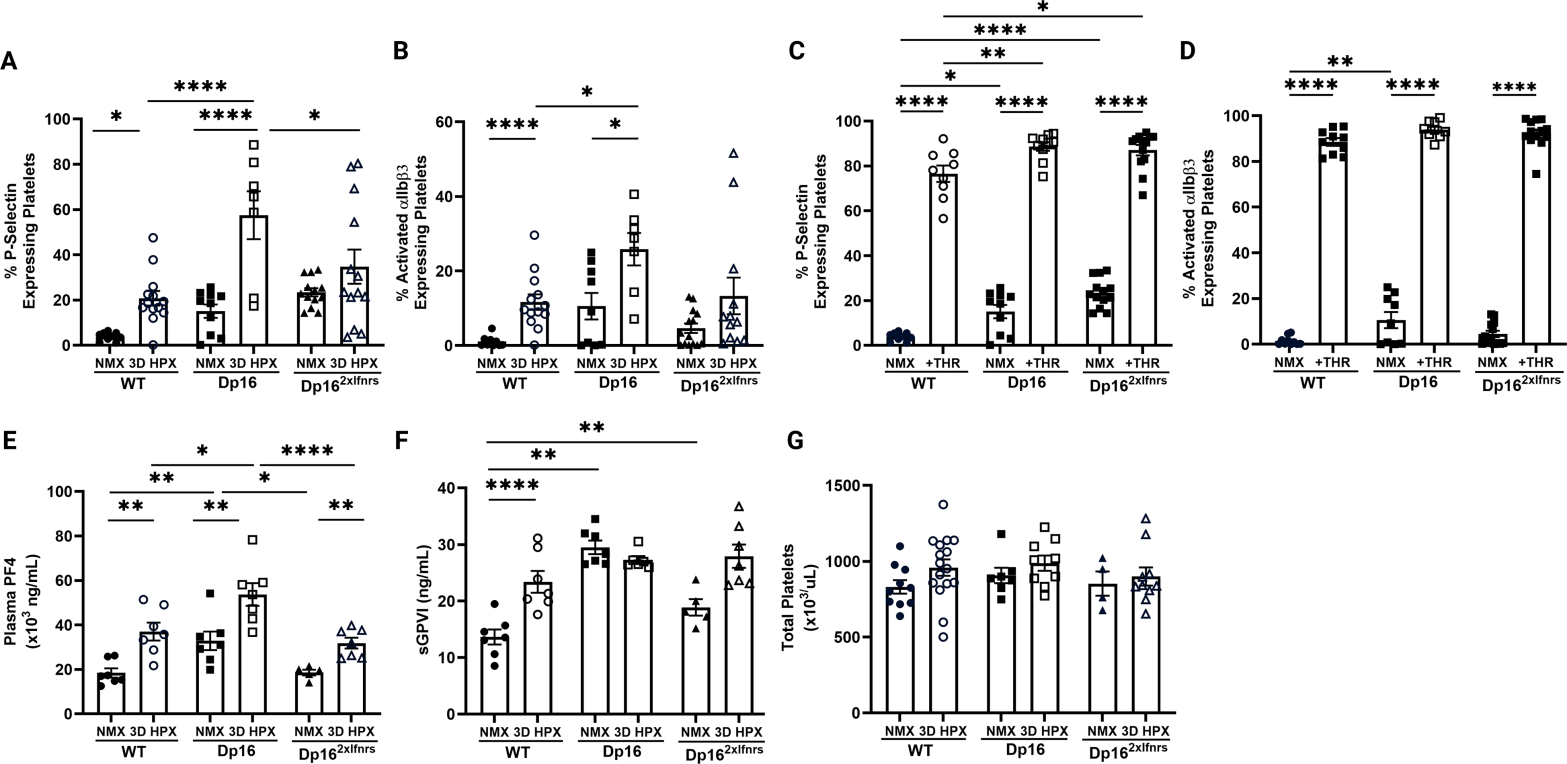
Platelet activation is increased and *Ifnr* copy-dependent in Dp16 mice. Platelet activation was measured by flow cytometry. *WT NMX, n=10; WT 3D HPX, n=13; Dp16 NMX, n=9; Dp16 3D HPX, n=7; Dp16^2xIfnrs^ NMX, n= 13; Dp16^2xIfnrs^ 3D HPX, n=13.* **(A, B)** Platelet P-selectin and αIIbβ3 (JONA) activation was increased at baseline and exacerbated by hypoxia in Dp16 mice and normalized in Dp16^2xIfnrs^ mice. **(C)** Activation potential was measured in platelets stimulated with Thrombin. *WT NMX and WT + THR, n=9; Dp16 NMX and Dp16 + THR, n=10; NMX, n=13; Dp16^2xIfnrs^ NMX + THR, n=13.* P-selectin was increased at baseline in Dp16 and Dp16^2xIfnrs^ mice. Thrombin-induced P-selectin expression was exacerbated at Dp16 and Dp16^2xIfnrs^ mice. **(D)** αIIbβ3 (JONA) activation was increased at baseline in Dp16 mice. Thrombin-induced αIIbβ3 (JONA) activation is similar between WT, Dp16, and Dp16^2xIfnrs^ mice. **(E)** Circulating PF4 was measured by plasma ELISA. *WT NMX, n=7; WT 3D HPX, n=7; Dp16 NMX, n=8; Dp16 3D HPX, n=7; Dp16^2xIfnrs^ NMX, n= 5; Dp16^2xIfnrs^ 3D HPX, n=7.* Plasma PF4 was increased at baseline and exacerbated in hypoxia in Dp16 mice. Baseline and hypoxia-induced plasma PF4 was attenuated in Dp16^2xIfnrs^ mice. **(F)** Soluble GPVI was measured by plasma ELISA. *WT NMX, n=7; WT 3D HPX, n=7; Dp16 NMX, n=8; Dp16 3D HPX, n=7; Dp16^2xIfnrs^ NMX, n= 5; Dp16^2xIfnrs^ 3D HPX, n=7.* sGPVI is increased in hypoxic WT mice. Dp16 and Dp16^2xIfnrs^ mice have elevated sGPVI at baseline compared to WT mice, which do not increase further in hypoxia. **(G)** Total platelet count was obtained using a hematologic analyzer. *WT NMX, n=10; WT 3D HPX, n=16; Dp16 NMX, n=7; Dp16 3D HPX, n=9; Dp16^2xIfnrs^ NMX, n= 4; Dp16^2xIfnrs^ 3D HPX, n=10.* Total circulating platelet count was similar in WT, Dp16, and Dp16^2xIfnrs^ mice under normoxic and hypoxic conditions. Normally distributed data were analyzed using two-way ANOVA with Tukey’s *post hoc* testing for **(A, C, G)**. Non-parametric data was analyzed using aligned rank transformation (ART) with Holm adjustment **(B, D–F)**. All data are expressed as mean ± SEM. Statistics: *p ≤ 0.05, **p<0.01, ***p<0.001, ****p<0.0001.

Platelets are increasingly recognized as central regulators of thromboinflammation, bridging coagulation and immune signaling in vascular disease (14, 16, 17, 20, 23, 25–28). We and others have shown that hypoxia activates platelets in both experimental models and human disease, contributing to vascular pathology through the release of inflammatory and vasoactive mediators (15, 16, 20, 28). At baseline, Dp16 mice exhibited increased platelet activation compared to WT controls, as demonstrated by elevated P-selectin expression and increased activation of integrin αIIbβ3 (**Figures 2A, B**). Hypoxia increased platelet activation in WT mice and this response was exaggerated in Dp16 mice. To determine whether this phenotype was dependent on IFNR gene dosage, we evaluated platelet activation in Dp16^2xIfnrs^ mice. Normalization of *Ifnr* copy number attenuated both baseline and hypoxia-induced platelet activation, restoring responses toward WT levels (**Figures 2A, B**). Consistent with these findings, agonist-induced P-selectin release was enhanced in Dp16 mice. Agonist-induced P-selectin release was also exacerbated in Dp16^2xIfnrs^ mice while agonist-induced αIIbβ3 activation was comparable across genotypes (**Figures 2C, D**).

### Circulating markers of platelet activation are increased in Dp16 mice and partially normalized by *Ifnr* copy number correction

To further characterize platelet activation, we measured circulating markers of platelet degranulation and activation. PF4, a chemokine predominantly stored in platelet α-granules, has been implicated in vascular remodeling and immune regulation within the pulmonary vasculature (9, 22, 30, 75–81). Plasma PF4 levels were increased at baseline in Dp16 mice compared to WT controls and further increased following hypoxia (**Figure 2E**). This response was attenuated in Dp16^2xIfnrs^ mice, consistent with IFN-dependent regulation. GPVI is a major platelet collagen receptor and has been implicated in platelet-mediated pulmonary vascular inflammation (75, 82). Soluble GPVI (sGPVI) was increased in hypoxic WT mice, while Dp16 mice exhibited elevated baseline levels that did not further increase with hypoxia (**Figure 2F**). Baseline sGPVI levels in Dp16^2xIfnrs^ mice were also elevated with no significant increase in hypoxia, suggesting differential regulation of GPVI shedding compared to α-granule release. Total circulating platelet counts were similar across genotypes and conditions (**Figure 2G**), indicating that differences reflect platelet activation rather than platelet number.

### Lung PF4 accumulation is increased in Dp16 mice and attenuated by *Ifnr* normalization despite similar platelet accumulation

**Figure 3:**
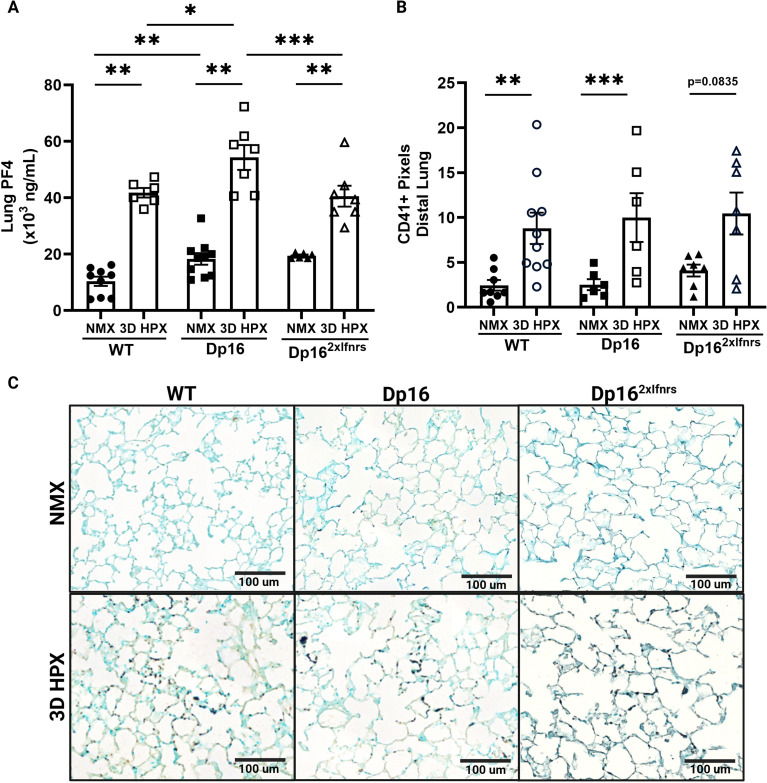
Lung PF4 accumulation is increased in Dp16 mice and attenuated by Ifnr normalization despite similar platelet accumulation. **(A)** Lung PF4 was measured by ELISA. *WT NMX, n=9; WT 3D HPX, n=6; Dp16 NMX, n=10; Dp16 3D HPX, n=7; Dp16^2xIfnrs^ NMX, n= 8; Dp16^2xIfnrs^ 3D HPX, n=7.* Lung PF4 was increased at baseline and exacerbated in hypoxic Dp16 mice. Baseline and hypoxia-induced exacerbation in lung PF4 was normalized in Dp16^2xIfnrs^ mice. **(B)** Lung platelets were assessed using IHC. *WT NMX, n=8; WT 3D HPX, n=10; Dp16 NMX, n=6; Dp16 3D HPX, n=6; Dp16^2xIfnrs^ NMX, n= 7; Dp16^2xIfnrs^ 3D HPX, n=7.* Platelets accumulated similarly in the distal lung of hypoxic WT, Dp16 mice, and Dp16^2xIfnrs^ mice. **(C)** Whole slide scans were obtained using the Leica Aperio VERSA brightfield scope (40x objective, 0.5-micron resolution). Representative images of CD41 staining in the distal lung; x20 magnification, scale bars = 100 μm. All images were acquired at identical magnification and processed using consistent scaling parameters; apparent differences reflect underlying changes in lung architecture rather than image scaling. Normally distributed data were analyzed using two-way ANOVA with Tukey’s *post hoc* testing for **(A)**. Non-parametric data was analyzed using aligned rank transformation (ART) with Holm adjustment **(B)**. All data are expressed as mean ± SEM. Statistics: *p ≤ 0.05, **p<0.01, ***p<0.001, ****p<0.0001.

We previously demonstrated that platelets are increased in the lungs of hypoxic WT mice and in patients with advanced PH (28, 32, 65, 83). Work by our group and others has demonstrated that the PF4 protein accumulates within platelets, in vascular tissue, and extracellularly in the hypoxic murine lung and in patients with PH, where it promotes inflammation and endothelial dysfunction (19, 22, 30, 32, 33, 84). Lung PF4 was increased at baseline in Dp16 mice and further elevated following hypoxia compared to WT controls (**Figure 3A**). This was attenuated in Dp16^2xIfnrs^ mice, indicating IFN-dependent regulation of platelet-derived inflammatory signaling. Despite these differences, platelet accumulation within the lung was similar across genotypes at baseline and increased comparably following hypoxia (**Figures 3B, C**). These findings suggest that increased PF4 reflects enhanced platelet activation and degranulation rather than increased platelet recruitment to the lung.

### Pulmonary hypertension phenotypes are differentially regulated in Dp16 mice

**Figure 4:**
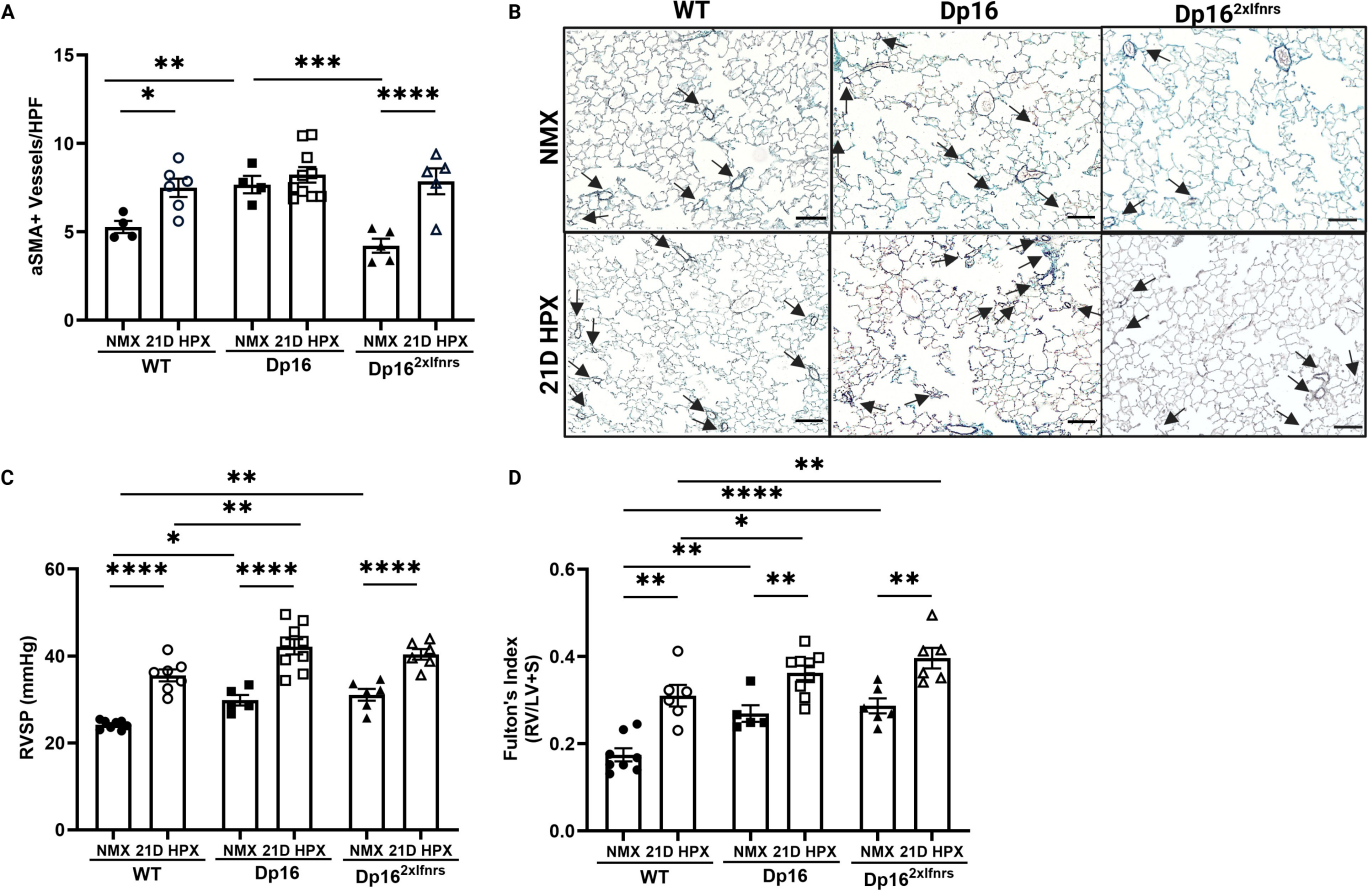
Pulmonary hypertension phenotypes are differentially regulated in Dp16 mice. **(A)** Pulmonary vascular remodeling quantified by small vessel muscularization in vessels <50 um in size. *WT NMX, n=4; WT 21D HPX, n=6; Dp16 NMX, n=4; Dp16 21D HPX, n=10; Dp16^2xIfnrs^ NMX, n=5; Dp16^2xIfnrs^ 21D HPX, n=5.* Small vessel muscularization was increased in hypoxic WT mice. Dp16 mice had elevated small vessel muscularization at baseline compared to WT mice, which did not increase further in hypoxia. Baseline small vessel muscularization is normalized in in Dp16^2xIfnrs^ mice. Hypoxia-induced muscularization of vessels <50 μm was similar between WT, Dp16, and in Dp16^2xIfnrs^ mice. **(B)** Representative aSMA staining of WT and Dp16 lungs exposed to normoxia (RA at Denver altitude) or 21-day hypoxia; x40 magnification, scale bars = 50 μm; black arrows = aSMA+ vessels <50um. All images were acquired at identical magnification and processed using consistent scaling parameters; apparent differences reflect underlying changes in lung architecture rather than image scaling. PH was assessed by RVSP and RVH. *WT NMX, n=8; WT 21D HPX, n=5; Dp16 NMX, n=5; Dp16 21D HPX, n=9; Dp16^2xIfnrs^ NMX, n=6; Dp16^2xIfnrs^ 21D HPX, n=6.* **(C)** RVSP assessed via direct closed-chest RV puncture. RVSPs were increased at baseline in Dp16 and Dp16^2xIfnrs^ mice. Hypoxia-induced RVSP was exacerbated in Dp16 mice and this exacerbation was prevented in Dp16^2xIfnrs^ mice. **(D)** RVH measured using Fulton’s index (ratio weight of R/LV+S). RVH was increased at baseline and exacerbated in hypoxia in Dp16 and Dp16^2xIfnrs^ compared to WT mice. Normally distributed data were analyzed using two-way ANOVA with Tukey’s *post hoc* testing for **(A, D)**. Non-parametric data was analyzed using aligned rank transformation (ART) with Holm adjustment **(C)**. All data are expressed as mean ± SEM. Statistics: *p ≤ 0.05, **p<0.01, ***p<0.001, ****p<0.0001.

We evaluated the degree of muscularization of small pulmonary vessels, a hallmark of pathological pulmonary vascular remodeling (1, 32, 85, 86). We assessed muscularization of small pulmonary vessels (<50 μm) as a measure of remodeling. Dp16 mice exhibited increased baseline small vessel muscularization compared to WT controls (**Figures 4A, B**). Hypoxia increased muscularization in WT mice, however, Dp16 mice did not demonstrate further increases, suggesting a pre-existing remodeling phenotype. Normalization of *Ifnr* copy number in Dp16*^2xIfnrs^* mice reduced baseline vascular remodeling to levels comparable to WT controls, indicating that this phenotype is IFN-dependent. Dp16*^2xIfnrs^* mice exhibit hypoxia-induced small vessel muscularization similar to WT mice. A key consequence of sustained pressure overload in the pulmonary circulation is elevated RV pressures and the development of RVH, an adaptive response to increased afterload (1, 2, 4, 5). To determine whether IFN signaling contributes to the development of pulmonary hypertension, we assessed right ventricular systolic pressure (RVSP) and right ventricular hypertrophy (RVH). Dp16 mice exhibited increased RVSP and RVH at baseline compared to WT controls, and both parameters were further increased following hypoxia (**Figures 4C, D**). Interestingly, normalization of *Ifnr* copy number restored hypoxia-induced RVSP to WT levels but baseline RVSP remained elevated. *Ifnr* normalization did not result in attenuation of baseline or hypoxia-induced RVH (**Figures 4C, D**).

## Discussion

**Figure 5:**
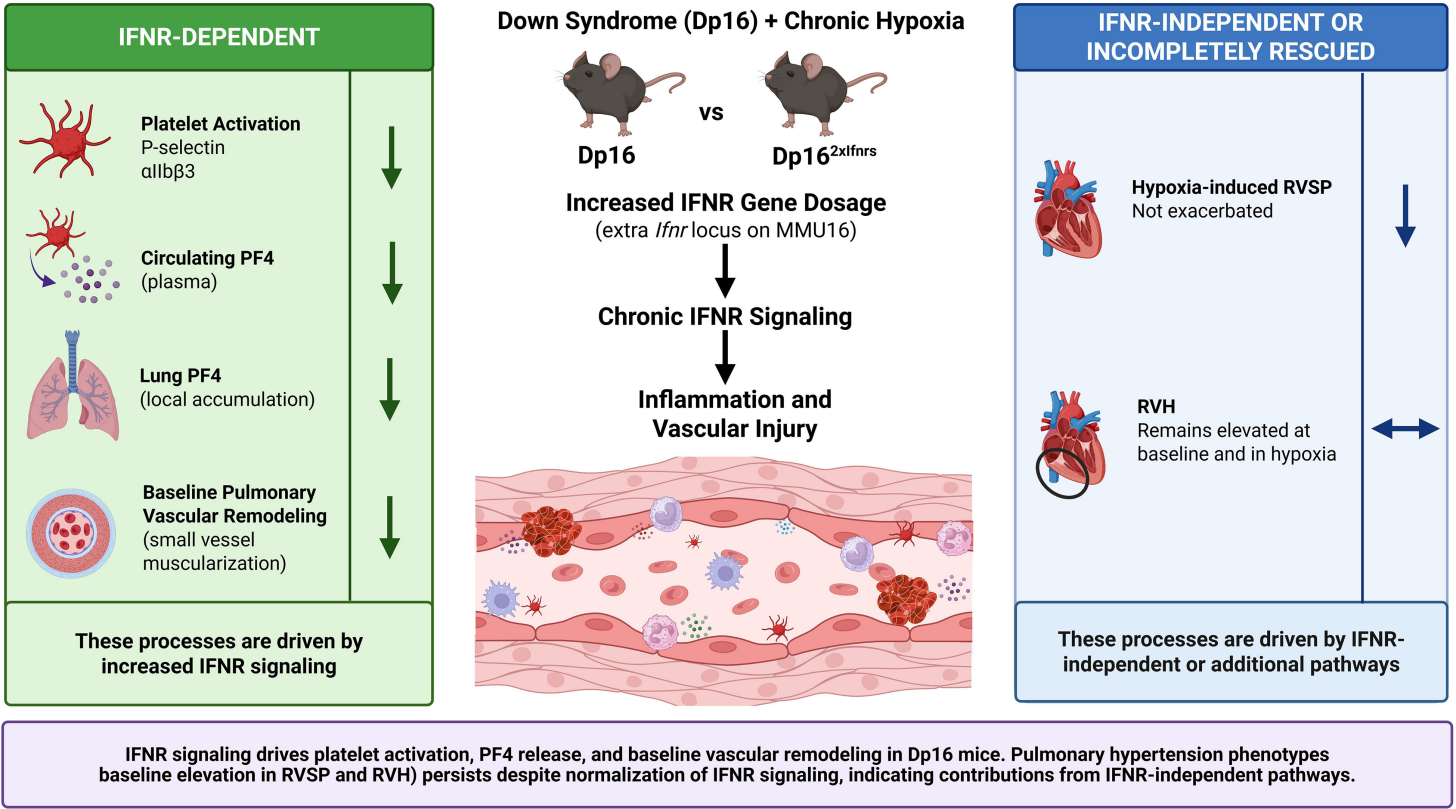
Summary model of IFNR-dependent and IFNR-independent mechanisms identified in this study. In the Dp16 mouse model, increased IFNR gene dosage promotes platelet activation and inflammatory signaling, as evidenced by increased platelet P-selectin, activated αIIbβ3, plasma PF4, and lung PF4. These phenotypes, along with baseline pulmonary vascular remodeling and hypoxia-induced RVSP exacerbation are attenuated by normalization of *Ifnr* copy number in Dp16^2xIfnrs^ mice, indicating IFNR dependence. In contrast, elevated baseline RVSPs persists despite *Ifnr* normalization, and RVH remains elevated at baseline and in hypoxia compared to WT controls, indicating incomplete rescue of cardiopulmonary phenotypes. Together, these findings support a model in which IFNR signaling contributes to platelet activation and structural vascular changes but is not sufficient to determine the full hemodynamic severity of pulmonary hypertension in Trisomy 21. Created in Biorender (https://BioRender.com).

T21 and PH are both characterized by persistent, dysregulated inflammation and PH is a common and significant comorbidity in individuals with T21 (48, 73, 87–89). In T21, *Ifnr* gene triplication drives a chronic interferonopathy that contributes to heightened immune signaling and may exacerbate pathways involved in PH development (57, 58, 90). We and others have demonstrated that circulating platelets are activated and that thrombosis is commonly observed in the lungs of patients with PH (15, 16, 18, 20, 91–93). Therefore, we hypothesized that persistent IFN activation in Dp16 mice contributes to platelet activation and vascular inflammation associated with pulmonary vascular remodeling. However, our data demonstrate that IFNR gene dosage exerts selective effects across platelet activation and cardiopulmonary phenotypes, rather than uniformly driving disease. We show, for the first time, that platelets are activated in patients with T21 and are more primed for activation, as evidenced by exacerbated responses to platelet agonists. Platelet activation was increased at baseline and further exacerbated by hypoxia in Dp16 mice, and this response was *Ifnr* copy-dependent. We also observed increased lung PF4 at baseline and in hypoxia in Dp16 mice, despite similar numbers of lung platelets, and this was attenuated in Dp16^2xIfnrs^ mice. Normalization of *Ifnr* copy number corrected the elevated baseline pulmonary vascular remodeling observed in Dp16 mice but did not alter hypoxia-induced remodeling, which was comparable across genotypes. In contrast, *Ifnr* copy normalization restored hypoxia-induced RVSP to WT levels, but baseline RVSP remained elevated in Dp16^2xIfnrs^ mice. RVH was elevated at baseline and following hypoxia in Dp16 and Dp16^2xIfnrs^ mice. Together, these results support a model in which interferon signaling primarily influences early inflammatory and structural processes, whereas the development of pulmonary hypertension and right ventricular adaptation is driven by additional IFNR-independent mechanisms. These findings are summarized schematically in **Figure 5**.

Our observation that platelets from individuals with T21 exhibit increased activation and heightened responsiveness to agonists, demonstrated by elevated surface expression of α-granule protein P-selectin and increased activation of integrin αIIbβ3, provides translational support for the Dp16 mouse as a relevant *in vivo* model for studying interferon-driven platelet biology. Consistent with these human findings, platelets from Dp16 mice displayed an increased baseline P-selectin and PF4 release and αIIbβ3 activation. Agonist-induced P-selectin release was exaggerated compared to WT controls, while αIIbβ3 activation remained similar between genotypes. These findings suggest that Dp16 platelets retain comparable aggregation potential in response to thrombin but may be predisposed toward enhanced α-granule secretion and may contribute to the immune dysregulation seen in individuals with T21. Hypoxia further exacerbated platelet activation in Dp16 mice relative to WT controls indicating that these differences are even more pronounced under physiological stress. Importantly, normalization of *Ifnr* copy number in the Dp16^2xIfnrs^ mouse rescued baseline and hypoxia-induced platelet activation, preventing exaggerated P-selectin release and αIIbβ3 activation. These results provide support for targeting this pathway in individuals with T21 as a strategy to restore immune homeostasis.

Supporting this interpretation, interferon signaling has well-established effects on hematopoiesis and megakaryopoiesis (51, 94, 95). Type I IFNs alter megakaryocyte maturation and α-granule composition, whereas IFN-γ can enhance platelet activation and platelet–leukocyte interactions (51, 54, 56, 94, 95). Lastly, we observed no differences in circulating platelet counts in individuals with T21 or Dp16 mice and their respective controls, indicating that the observed effects are attributable to altered platelet function rather than differences in platelet number. We speculate that the elevated PF4 levels observed in Dp16 mice are not due to increased platelet numbers but reflect enhanced platelet activation and degranulation within the pulmonary microenvironment, consistent with what was observed in the circulation. Together, these findings indicate that platelet hyperactivation represents an intrinsic feature of the Dp16 mouse model and supports a mechanism whereby interferon pathway dysregulation in T21 promotes baseline platelet priming and thromboinflammatory signaling.

PF4 has known immunomodulatory and antiangiogenic properties (9, 22, 30, 75–81). Persistent type I IFN signaling is known to promote endothelial injury, which may enhance PF4 consumption or redistribution to inflamed vascular compartments (55, 96). This pattern is consistent with redistribution rather than overproduction, potentially reflecting local PF4 deposition at sites of endothelial activation or immune-complex formation where PF4 acts to limit endothelial injury (97). Such redistribution could contribute to localized inflammation and vascular remodeling. Normalization of *Ifnr* copy number prevented baseline and hypoxia-induced exaggeration of circulating and lung PF4 observed in Dp16 mice. Notably, PF4 can engage Toll-like receptor 9 (TLR9) and enhance type I IFN production suggesting a potential feedback loop that could amplify pulmonary inflammation (98). GPVI is a major platelet collagen receptor and has been implicated in platelet-mediated pulmonary vascular inflammation and remodeling, including smooth muscle proliferation and fibrotic responses to injury (82, 99–101). sGPVI is elevated at baseline in Dp16 mice, and while baseline sGPVI levels in Dp16^2xIfnrs^ mice trended toward WT levels in parallel with normalization of baseline pulmonary vascular remodeling, these differences did not reach statistical significance. Along with prevention of baseline pulmonary vascular remodeling in Dp16^2xIfnrs^ mice, these observations raise the possibility that GPVI-mediated signaling may contribute to vascular remodeling in this context, the current data are insufficient to establish a mechanistic link. Further studies will be required to determine whether GPVI-dependent pathways play a functional role in pulmonary vascular remodeling in T21.

Despite these IFN-dependent effects on platelet activation, PF4 signaling, and pulmonary vascular remodeling, *Ifnr* copy normalization did not fully prevent PH in this model. Pulmonary vascular remodeling, RVSP and RVH represent distinct but interconnected components of cardiopulmonary disease. These findings indicate that IFNR signaling contributes to baseline structural vascular abnormalities but does not determine the hemodynamic burden or cardiac remodeling associated with pulmonary hypertension. We speculate that additional genes located on human chromosome 21 and mouse chromosome 16 may contribute to the regulation of cardiopulmonary phenotypes in this model (37, 45).

Emerging evidence highlights the importance of right ventricular–intrinsic inflammatory and metabolic processes in determining PH progression and outcomes (5, 13, 102). In T21 and the Dp16 mouse, increased gene dosage of regulators of chromatin accessibility, oxidative stress, lymphangiogenesis, and inflammatory signaling may collectively influence both vascular and cardiac development, as well as responses to stress. *HMGN1* has been implicated in altered chromatin structure and amplification of inflammatory transcriptional programs, potentially enhancing immune activation within both the pulmonary vasculature and RV myocardium (45, 103–106). In parallel, *RCAN1*, a regulator of calcineurin–NFAT signaling, has been linked to maladaptive cardiac hypertrophy, vascular smooth muscle proliferation, and lymphatic abnormalities (107–112). Increased dosage of *SOD1* contributes to the oxidative stress phenotype of T21, where disproportionate superoxide dismutation promotes accumulation of reactive oxygen species, resulting in redox imbalance, reduced nitric oxide bioavailability, endothelial dysfunction, and impaired lymphangiogenesis (113, 114). Finally, *DYRK1A*, a dosage-sensitive kinase, modulates inflammatory and stress-response signaling pathways and has been implicated in cellular proliferation and cardiac development, suggesting a role in both vascular remodeling and RV adaptation to stress (115–117). Together, these pathways may converge to drive pulmonary vascular remodeling and RV dysfunction through coordinated effects on endothelial activation, inflammatory signaling, and cardiac stress adaptation. In this context, interferon-driven platelet activation likely represents an amplifying thromboinflammatory axis within a broader, multifactorial disease network, rather than a singular causal driver of PH.

Despite the strengths of this study, several limitations should be considered in the interpretation of this data. While IFN-dependent platelet activation, PF4 accumulation, and pulmonary vascular remodeling are strongly associated in this study and by prior literature, these data do not establish causality. It remains unclear whether platelet activation acts as a driver, amplifier, or biomarker of pulmonary vascular pathology in T21. Establishing causality will require platelet-specific experimental approaches, including platelet depletion, GPVI blockade, or platelet-specific manipulation of IFNR signaling. Future studies incorporating these strategies will be essential to define the mechanistic contribution of platelets to pulmonary hypertension pathogenesis in this model. Additionally, hemodynamic assessment was limited to RVSP and RVH. Additional measures of RV function and structural and functional cardiac studies would provide a more comprehensive evaluation of disease severity. Lastly, human platelet analyses were not linked to PH status or disease severity, as the current cohort was not designed or powered for clinical phenotyping. Accordingly, these findings should be interpreted as proof-of-principle for altered platelet activation in T21. Ongoing studies are focused on expanding recruitment to include larger, clinically characterized cohorts of individuals with T21 both with and without PH, incorporating comprehensive hemodynamic, imaging, and biomarker data to define the relationship between platelet activation and cardiopulmonary outcomes.

Collectively, our findings demonstrate that platelet activation is exaggerated in individuals with T21 and in the Dp16 mouse model, supporting the presence of a platelet-driven proinflammatory phenotype. These results further validate the Dp16 model as a relevant *in vivo* system for interrogating interferon-mediated mechanisms linking platelet activation, vascular inflammation, and cardiopulmonary disease in Down syndrome. Pulmonary vascular remodeling was attenuated by normalization of *Ifnr* copy number without leading to complete attenuation of PH, supporting a multifactorial model in which interferon-driven inflammation represents an amplifying axis rather than a singular driver of disease. This incomplete rescue highlights the need for further investigation of cardiopulmonary physiology in this model, including right ventricular function and RV–pulmonary artery coupling, as well as additional IFNR-independent mechanisms contributing to disease severity. Importantly, therapeutic strategies that reduce IFN-driven vascular inflammation may modulate platelet activation and structural vascular components of disease, even if they are insufficient to reverse PH. Consistent with this concept, emerging evidence that JAK–STAT inhibition can attenuate inflammatory signaling in T21, supporting future investigation of therapies that target IFN-mediated thromboinflammation (74, 118).

## Data Availability

The raw data supporting the conclusions of this article will be made available by the authors, without undue reservation.

## Supplementary material

Supplementary Figure 1 Novel Dp16 model of Down Syndrome. **(A)** Female WT^1xIfnrs^ were intercrossed with Dp(16)1Yey/+ (Dp16) males to generate WT, Dp16, and Dp16^2xIfnrs^ mice. **(B)** There were no weight differences across strains. Created in Biorender (https://BioRender.com).

Supplementary Figure 2 Experimental design of the chronic hypoxia model of pulmonary hypertension. WT, Dp16, and Dp16^2xIfnrs^ mice were maintained at Denver altitude or exposed to hypobaric hypoxia (PB = 380 mmHg, FiO_2_ = 10%) for 3 or 21 days. The 3-day timepoint was selected to assess early inflammatory responses, while the 21-day timepoint was selected to assess established pulmonary hypertension phenotypes. Created in Biorender (https://BioRender.com).
