# Supplementary figures and images for: Interferon receptor gene dosage differentially regulates hypoxia-induced platelet activation and pulmonary hypertension in down syndrome
Source: Front Immunol. 2026 May 22;17:1832057. doi: 10.3389/fimmu.2026.1832057 (PMC13236561; doi:10.3389/fimmu.2026.1832057)

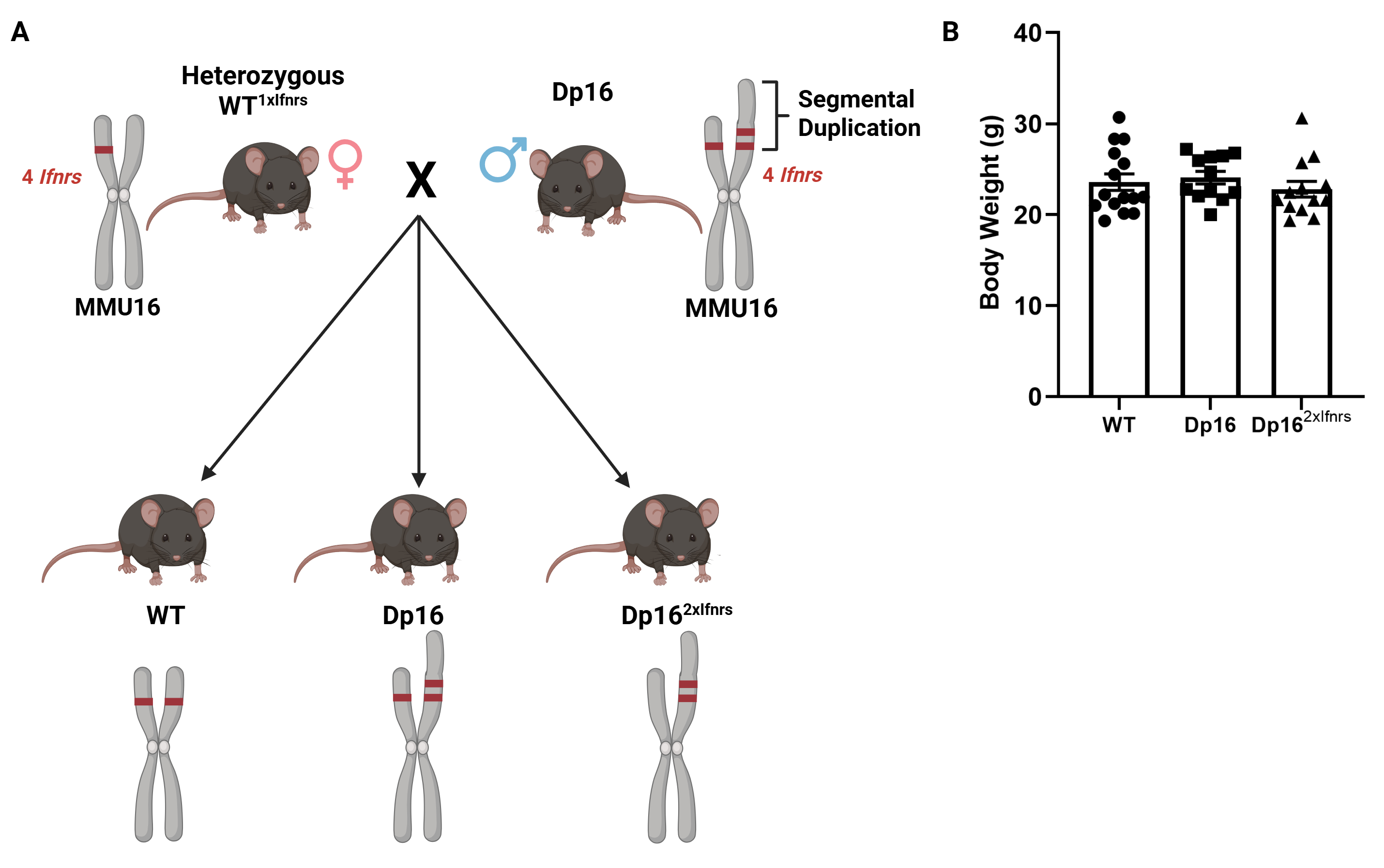

Supplement: Supplementary Figure 1 — Novel Dp16 model of Down Syndrome. (A) Female WT1xIfnrs were intercrossed with Dp(16)1Yey/+ (Dp16) males to generate WT, Dp16, and Dp162xIfnrs mice. (B) There were no weight differences across strains. Created in Biorender (https://BioRender.com). [file Image1.jpeg]

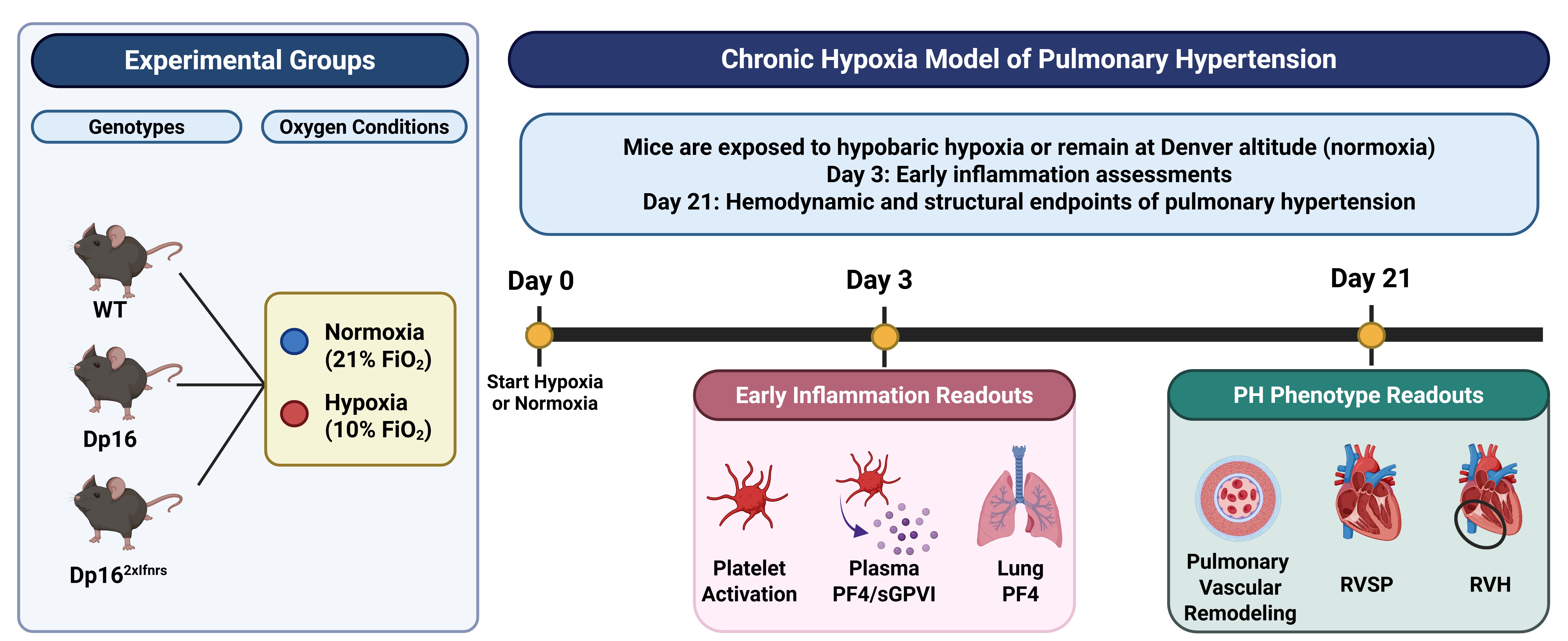

Supplement: Supplementary Figure 2 — Experimental design of the chronic hypoxia model of pulmonary hypertension. WT, Dp16, and Dp162xIfnrs mice were maintained at Denver altitude or exposed to hypobaric hypoxia (PB = 380 mmHg, FiO2 = 10%) for 3 or 21 days. The 3-day timepoint was selected to assess early inflammatory responses, while the 21-day timepoint was selected to assess established pulmonary hypertension phenotypes. Created in Biorender (https://BioRender.com). [file Image2.jpeg]
